# Detection of Beijing strains of MDR *M. tuberculosis* and their association with drug resistance mutations in *kat*G, *rpo*B, and *emb*B genes

**DOI:** 10.1186/s12879-020-05479-5

**Published:** 2020-10-14

**Authors:** Anamika Gupta, Pallavi Sinha, Vijay Nema, Pramod K. Gupta, Pampi Chakraborty, Savita Kulkarni, Nalin Rastogi, Shampa Anupurba

**Affiliations:** 1 Department of Microbiology, Institute of Medical Sciences, Banaras Hindu University, Varanasi, 221 005 India; 2 Division of Molecular Biology, National AIDS Research Institute, 73 G MIDC Bhosari, Pune, 411026 India; 3 Laboratory Nuclear Medicine Section, Isotope Group, Bhabha Atomic Research Centre C/o T.M.H. Annexe, Parel, Mumbai, 400012 India; 4 WHO Supranational TB Reference Laboratory, TB & Mycobacteria Unit, Institute Pasteur de Guadeloupe, Abymes, Guadeloupe France

**Keywords:** Beijing genotype, Rv2820, Spoligotyping, Drug resistant-TB, Transmission, Tuberculosis

## Abstract

**Background:**

Molecular epidemiological studies of *Mycobacterium tuberculosis* (MTB) are the core of current research to find out the association of the *M. tuberculosis* genotypes with its outbreak and transmission. The high prevalence of the Beijing genotype strain among multidrug resistance (MDR) TB has already been reported in various studies around India. The overall objective of this study was to detect the prevalence of Beijing genotype strains of MDR *M. tuberculosis* and their association with the clinical characteristics of TB patients.

**Methods:**

In this study 381 *M. tuberculosis* clinical isolates were obtained from sputum samples from 2008 to 2014. The multiplex-PCR and Spoligotyping (*n* = 131) methods were used to investigate the prevalence of the Beijing genotype strain by targeting the Rv2820 gene and their association with drug resistance and clinical characteristics of TB patients. The drug susceptibility testing of first-line anti-TB drugs was performed by using the proportion method and MGIT960. A collection of isolates having Beijing and non-Beijing strains were also characterized to see if Beijing genotype strains had a higher rate of mutations at codons 516, 526 and 531 of the 81-bp region of the *rpoB* gene, codon 315 of the *katG* gene, and codon 306 of the *embB* gene.

**Results:**

The sensitivities and specificities of multiplex-PCR assay compared to that of standard Spoligotyping was detected to be 100%. Further, we observe that the multi drug-resistance was significantly associated with Beijing genotype strains (*p* = 0.03) and a strong correlation between Beijing genotype strains and specific resistance mutations at the *katG*315, *rpoB*531, and *embB*306 codons (*p* = < 0.0001, < 0.0001 & 0.0014 respectively) was also found.

**Conclusions:**

This rapid, simple, and cost-effective multiplex PCR assay can effectively be used for monitoring the prevalence of Beijing genotype strains in low resource settings. Findings of this study may provide a scientific basis for the development of new diagnostic tools for detection and effective management of DR-TB in countries with a higher incidence rate of Beijing genotype strains.

## Background

Beijing genotype of *M. tuberculosis* (MTB) strain attracted expert’s attention due to its association with multidrug resistance (MDR) and disease outbreaks [1]. Justifications for the rapid dissemination of Beijing genotype strain have included its putative elevated virulence, enhanced transmissibility, greater mutability, ability to escape from BCG vaccine-induced immunity, and its skill to acquire multidrug resistance [2–5]. Information about the transmission of Beijing-genotype MTB strain circulating in North India is lacking. Therefore, as a part of our overall objective we have attempted to assess the prevalence of Beijing genotype MTB strain and its relation to drug resistance and clinical characteristics of TB patients in North India.

An extensive review of the mechanism behind successful emergence of the Beijing genotype strain has revealed a higher frequency of mutation at the codon 315 of *katG* gene among MTB Beijing genotype strains [6]. However, the association is less clear for mutations at codons 516, 526, and 531 of the *rpoB* gene, and at the codon 306 of the *embB* gene [5]. We characterized a collection of MTB patient isolates to see if the Beijing genotype strain had a higher rate of mutations at the *katG*315 codon, the *rpoB516, rpoB526,* and *rpoB531* codons of RRDR region which accounts for > 95% of resistance to rifampicin (RIF), and *embB*306, which is responsible for resistance to ethambutol (EMB) [7, 8]. Further, we also have evaluated a multiplex PCR against standard spoligotyping for the rapid and cost-effective detection of Beijing genotypes of MTB strains.

## Methods

### Setting

Banaras Hindu University (BHU) hospital is a tertiary-care hospital with vast catchment area, being the only tertiary care hospital in Eastern Uttar Pradesh (UP), situated in North Central India, providing medical cover to over 200 million population of Eastern UP, Western Bihar and adjoining areas of Madhya Pradesh. It’s a tertiary care center with a referral bias towards non-responding cases.

### Study subjects

TB patients, age range 6–90 years, with or without any other additional complications such as HIV-seropositivity, have been included in this study. Other details were received from the medical files and compliance charts. Treatment of TB patients was done according to the standard short-course chemotherapy under DOTS guidelines [9]. The written informed consent was obtained from all participants.

### Collection of samples

Four thousand seventy nine sputum specimens were collected during 2008–2014, from the patients of pulmonary tuberculosis, from three TB – centers and analyzed for culture positivity to *Mycobacterium tuberculosis*.

### Mycobacterial isolates

A total of 381 MTB isolates were isolated from 4079 TB suspected specimens collected over 6 years (2008 to 2014) from TB patients attending different health care centers and BHU hospital of Varanasi, UP, India.

### Drug susceptibility testing

A total of 381 MTB isolates were subjected to DST according to the standard proportion method (PM) and commercial MGIT960 system against RIF, INH, streptomycin (STR), and EMB [10]. H37Rv (ATCC 27294) and known RIF, INH, and EMB mono-resistant strains were used as negative and positive controls respectively.

### HIV testing

Three rapid HIV test kits based on different antigens/principles were used according to NACO (National AIDS Control Organization) guidelines for the detection of HIV in patients with TB [11].

### Multiplex-allele specific-PCR (MAS-PCR)/DNA sequencing for detection of RIF, INH and EMB resistance determinants

DNA was isolated as described by van Embden et al and a two-step MAS-PCR assay was performed to detect mutations at *rpoB* codons (516, 526, and 531), *katG* codon 315 and *embB* codon 306 [12–14]. Automated sequencing was performed using the BigDye Terminator kit v3.1 (Applied Biosystems, USA) as per manufacturer’s protocol. DNA sequence analysis was carried out for 61 strains. Further comparisons of *rpoB* (*n* = 128), *katG* (*n* = 128) and *embB*306 (*n* = 27) were carried out with SeqScape® v2.5 software.

### Spoligotyping

Out of 381 MTB isolates, spoligotyping was performed for 131 isolates according to the standard method of Kamerbeek et al. [15]. The spoligotypes were compared with those contained in the international database SITVIT2, an updated variant of the previously released SpolDB4 database [16]. Isolates of the Beijing genotype were defined by showing hybridization to at least three of the nine spacers within 35–43 and showing the absence of hybridization to spacers 1 to 34. Some Beijing strains lack one or more of the nine signature spacers, due to asymmetrical insertions of IS6110 (Table 3).

### Multiplex-PCR for the identification of Beijing genotypes of MTB targeting Rv2820 gene

**Fig. 1:**
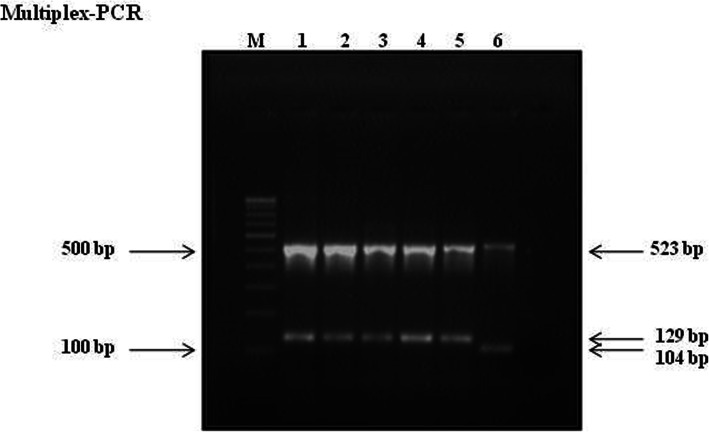
Agarose gel electrophoresis results of multiplex- PCR products on a 3% agarose gel: Lanes 1 to 5 showing Beijing strains (129 & 523 bp fragments); lane 7 showing a non-Beijing strain (104 & 523 bp fragments); lane M: 100-bp DNA ladder (Bangalore Genei)

To differentiate between Beijing and non-Beijing strains of 381 MTB isolate, three sets of PCR primers were used (Supplementary Table 1 [17, 18]). These were based on the information given by Warren et al. that the region covering genes Rv2816 to Rv2819 and portion of Rv2820 is deleted in all Beijing strains of MTB [19]. For multiplex PCR, 10 μl master mix was containing 0.1 U of DyNAzyme EXT (Finnzymes Oy, Espoo, Finland), 250 μM of each dNTP, 2.0 mM MgCl2, 5 μM of each primer, and 1 μl (10 ng) of template DNA in 1× EXT buffer. Reaction conditions included 96 °C for 4 min, followed by 35 cycles of 96 °C for 45 s, 55 °C for 45 s, and 72 °C for 1 min, and a final extension at 72 °C for 5 min. The amplified products were analyzed by electrophoresis on a 3% agarose gel. Beijing strains of MTB will produce one 523-bp internal control amplified product and one 129-bp product while non-Beijing strains will produce the 523-bp and a104-bp product (Fig. 1).

### Statistical analysis

Results were analyzed with the SPSS software (version 16.0.0) (SPSS Inc., Chicago, IL, USA). The features of the two groups were compared using the Z-test and of three groups by the chi-square (χ2) test, for statistical significance assessment.

## Results

### Patients

A total of 381 MTB isolates were isolated. Seventy-six (19.95%) isolates belong to Beijing genotype among which 45 (11.81%) were multidrug-resistant. Beijing genotype strains were significantly more common in women (*p* = 0.01) than in men (Table 1).

**Table 1:** Bivariate analysis for selected characteristics in patients with Beijing and non-Beijing genotypes of *M.tb* of North Central India

Parameters	Beijing genotype, n (%)	Non- Beijing genotypes, n (%)	Odds Ratio (OR)	***p***-value*	95% CI
**Sex**					
Male (250)	41 (53.95)	211 (69.18)	0.522	0.01	0.312–0.871
Female (131)	35 (46.05)	94 (30.82)			
**Age group**					
< 15 (11)	4 (5.26)	7 (2.3)	2.295	0.1	0.650–8.106
15–44 (290)	58 (76.32)	233 (76.39)			
≥ 45 (80)	14 (18.42)	65 (21.31)	1.155	0.6	0.606–2.203
**HIV Status**					
Sero positive (38)	8 (10.53)	30 (9.83)	1.078	0.8	0.473–2.458
Sero negative (343)	68 (89.47)	275 (90.16)			
**Site of TB**					
Pulmonary (368)	73 (96.05)	295 (96.72)	0.824	0.7	0.221–3.073
Extra-pulmonary (13)	3 (3.95)	10 (3.28)			
**History of TB**					
New cases (247)	39 (51.32)	202 (66.23)	0.537	0.01	0.323–0.894
Previously treated (134)	37 (48.68)	103 (33.77)			
**Drug resistance**					
Any drug resistance (194)	50 (65.79)	144 (47.21)	2.15		1.272–
Susceptible to all (R + H+ S + E) (187)	26 (34.21)	161 (52.79)		0.004	3.633
MDR (121)	45 (59.21)	76 (24.92)	0.586	0.032	0.359–0.956
Total Cases (381)	76 (19.95)	305 (80.05)	–	–	–

*A *p* value of < 0.05 was considered statistically significant

### Strains

Of the 32 strains that were Beijing genotype as defined by Spoligotyping (total Beijing genotype strains are 76), 30 had all nine characteristic spacers 35–43, corresponding to the shared type SIT1 as defined in SITVIT2. In the remaining two isolates, one isolate had the last six spacers while another had spacers 37–43 and spacer 35 correspondings to SIT250 and SIT621 respectively (Table 2).

**Table 2:** Description of Beijing clades found in this study as determined by spoligotyping (*n* = 32, 24.43%)

SIT	Spoligotype Description	Octal Number	Clade	Number (%) in study
1	□□□□□□□□□□□□□□□□□□□□□□□□□□□□□□□□□□■■■■■■■■■	000000000003771	Beijing	30 (22.90)
250	□□□□□□□□□□□□□□□□□□□□□□□□□□□□□□□□□□□□□■■■■■■	000000000000371	Beijing	1 (0.76)
621	□□□□□□□□□□□□□□□□□□□□□□□□□□□□□□□□□□■□■■■■■■■	000000000002771	Beijing	1 (0.76)

### Drug resistance

Among 76 Beijing strains, 47 were RIF resistant, 50 were resistant to INH, and 45 were found to be MDR. Thirty-seven strains were resistant to STR and 40 were EMB resistant (Supplementary Table 2). While 50 (65.79%) and 45 (59.21%) of the Beijing strains were any drug-resistant and MDR respectively, 144 (47.21%) and 76 (24.92%) of 305 non-Beijing strains were any drug-resistant and MDR during the same period representing a statistically significant difference (*p*-value 0.004 & 0.032 respectively) (Table 1).

### Multiplex-PCR Vs Spoligotyping

Multiplex PCR was performed for all 381 isolates and spoligotyping was performed in 131 (of 381) isolates. When statistically compared, the results of all 131 isolates were the same for the detection of Beijing and non-Beijing isolates with both the methods. The sensitivities and specificities of multiplex-PCR assay compared to that of standard Spoligotyping was observed to be 100%.

### Genotyping for RIF, INH and EMB resistance mutations

A mutation in *katG* gene, showing a nucleotide substitution at codon 315 from AGC to ACC, founded in 43/50 (86%) of the Beijing strains with phenotypic INH resistance. Resistance mutations at the *rpoB* gene were found in 45/47 (95.74%) Beijing strains with phenotypic RIF resistance. A mutation at *rpoB* codon 531 was most common (*n* = 29; 61.70%). Of 44 phenotypically EMB resistant isolates, 21 had mutations at *embB* codon 306 (Table 3). Compared to the wild-type (wt) isolates, the isolates with the *katG*315Thr and *rpoB*531Leu mutations were most frequent (86 and 61.70% respectively) in Beijing genotypes (Table 3). Mutations at *katG*315, *rpoB*531, and *embB*306 were more common among Beijing genotype strains, and these associations were observed to be statistically significant (*p* = < 0.0001, < 0.0001 and 0.0014 respectively) (Table 4).

**Table 3:** Drug-resistant Beijing strains of *M.tb* with different genetic mutation* (*n* = 76)

Gene	MDR strains, ***n*** = 45	RIF resistant strains, ***n*** = 47	INH resistant strains, ***n*** = 50	EMB resistant strains, ***n*** = 44
*kat*G				
315Thr	41	–	43	–
Others/wt	4	–	7	–
*rpo*B				
516Leu or Trp	3	3	–	–
526Arg,Tyr,Asp,Gly or Asn	4	5	–	–
531Leu or Trp	28	29	–	–
526Arg, Tyr.... + 531Leu or trp	6	6	–	–
516Leu or Trp +531Leu or Trp	2	2	–	–
Others/wt	2	2	–	–
*emb*B				
306Val	13	–	–	13
306Ile	8	–	–	8
Others/wt	24	–	–	23

*Mutation detection was done by Multiplex-allele specific-PCR; MDR #: Resistant to at least INH and RIF with or without resistance to any other anti-tubercular drug. *INH* isoniazid, *RIF* rifampicin, *STR* streptomycin, *EMB* ethambutol

**Table 4:** Association between *M.tb* Beijing genotype and drug-resistance gene mutations

Gene	Beijing genotype (***n*** = 76)	Non-Beijing genotypes (***n*** = 305)	OR (95% CI)	***p***-Value^*^
*kat*G315	43 (56.58%)	73 (23.93%)	4.14 (2.45 – 6.99)	< 0.0001
*rpo*B531	29 (38.16%)	49 (16.07%)	3.22 (1.85 – 5.61)	< 0.0001
*emb*B306	21 (27.63%)	38 (12.46%)	2.68 (1.46–4.92)	0.0014

*A *p* value of < 0.05 was considered statistically significant

## Discussion

Beijing genotype strain was first isolated in Beijing, China in 1995 and now it has been predominantly found in various parts of the world [20, 21]. A systematic review and meta-analysis on the prevalence of the Beijing genotype strain in world population revealed that its prevalence was 44.7% in Asia, 27.9% in Europe, 12.5% in Africa, and 8.9% in America [22]. In India, the Beijing genotype strain is present in a low percentage throughout the country including a previous study in our region [23–25] however we observed a comparatively higher incident rate of the Beijing genotype strain (19.95%, 76/381). Possible reason behind this discrepancy could be the low number of MTB strains studied in the previous study in our region.

We found a 100% agreement between multiplex-PCR targeting the Rv2820 gene and standard spoligotyping for the detection of Beijing genotype strains of MTB. Similar results were also observed by Sun et al and Sherafat et al [26, 27].

In an extensive review based on the data available from 49 studies in 35 countries on Beijing genotype MTB strain and drug resistance four different patterns of Beijing genotype strains were suggested worldwide but our study population represents a different group; endemic and associated with drug resistance which is now a common pattern for some other countries too [5, 28, 29].

The worldwide emergence of Beijing genotype strains and their association with multidrug-resistance substantiates the fact that they possess strong capability to acquire drug resistance. This is confirmed by a study, in which 14 drug-resistant clinical isolates of the Beijing genotype were sequenced by whole-genome sequencing to understand the evolution of drug resistance and results showed that the drug resistance among these isolates appeared to be acquired, not clonally derived [30]. Our study observed the similar drift, with 33/50 (66%) drug resistant-Beijing genotype strains and 29/45 (64.44%) MDR-Beijing genotype strains from acquired drug-resistant TB patients.

The higher rate of mutations at *rpoB*531, *katG*315, and *embB*306 codons among MTB, Beijing strains provides further evidence that drug resistance may contribute to the global predominance of Beijing genotype strains. In the present study, mutations at codons *katG*315, *rpoB*531 and *embB*306 were more common among Beijing genotype strains than in non-Beijing genotype strains of MTB (*p* = < 0.0001; < 0.0001; 0.0014) (Table 4). Similar findings were also observed in other studies [6, 31–34]. On the basis of our results we consider that recent transmission of Beijing genotype strains could be the driving force of such a high prevalence of the *katG*315, *rpoB*531 mutations. Another explanation could be; mutations in putative mutator genes have been found in Beijing genotypes, which results in an altered DNA repair and an increased mutation adaptability rate [28].

The strong association of Beijing genotype strain and *katG*, *rpoB,* and *embB* mutations among treated cases of TB, reflects the considerable transmission of MDR- Beijing genotype strains in our region which suggests the need of urgent change in the quality of chemotherapy applied in the community.

## Conclusions

In this study, we have successfully evaluated a simple, rapid, robust, and cost-effective multiplex PCR assay for the detection of Beijing genotype of *M. tuberculosis* strains by targeting Rv2820 gene. Study outcomes may provide a scientific basis for the development of new DR-TB diagnostic tools for effective management and control of DR-TB in countries with a higher incidence rate of Beijing genotype strains. Further, a recruitment of large number of representative samples for a systematic study is needed to elucidate the role of Beijing genotype strain in the transmission of TB and their association with DR-TB in the region. Additional studies using MIRU-VNTRs over an extended period of time can be done to fully understand the epidemiology of TB and its transmission dynamics in our region and all over India.

## Supplementary information

**Additional file 1.**

**Additional file 2.**

**Additional file 3.**

## Data Availability

The datasets used and analyzed during the current study available from the corresponding author on reasonable request.

## Abbreviations

MTB: *Mycobacterium tuberculosis*
DR-TB: Drug-resistant tuberculosis
RRDR: Rifampicin resistance determining region
MDR: Multidrug resistance
RIF: Rifampicin
EMB: Ethambutol
STR: Streptomycin
INH: Isoniazid
PM: Proportion method
BHU: Banaras Hindu University
UP: Uttar-Pradesh
