# Supplementary material for: Detection of Beijing strains of MDR M. tuberculosis and their association with drug resistance mutations in katG, rpoB, and embB genes
Source: BMC Infect Dis. 2020 Oct 14;20:752. doi: 10.1186/s12879-020-05479-5 (PMC7557036; doi:10.1186/s12879-020-05479-5)
Supplement: Supplementary file 1 — Additional file 1. [file 12879_2020_5479_MOESM1_ESM.doc]

**Supplementary Table 1**

Details of primers

| **Primer/Target** | **Sequence (5’-3’)** | **Size (bp)** | **Containing** | **Reference (Primers)** | **Annealing T (^0^C)** | **Primer (µM)** |
| --- | --- | --- | --- | --- | --- | --- |
| IS59/IS6110 | GCGCCAGGCGCAGGTCGATGC | 523 | Part of IS6110 | Plikaytis et al., 1994 | 55 | 5 |
| IS60/IS6110 | GATCAGCGATCGTGGTCCTGC |  |  |  |  |  |
| BjF/Beijing genotype | CTCGGCAGCTTCCTCGAT | 129 | 3′ end of IS6110 and the 5′ end of the Rv2820 gene | Hillemann et al., 2006 |  |  |
| BjR/Beijing genotype | CGAACTCGAGGCTGCCTACTAC |  |  |  | 55 | 5 |
| nBjF/non-Beijing genotype | AAGCATTCCCTTGACAGTCGAA | 104 | Rv2819 gene of non-Beijing strains of MTB |  | 55 | 5 |
| nBjR/non-Beijing genotype | GGCGCATGACTCGAAAGAAG |  |  |  |  |  |

Abbreviation used: MTB- *Mycobacterium tuberculosis*; F-forward, R-Reverse, T-temperature
