# Supplementary material for: Detection of Beijing strains of MDR M. tuberculosis and their association with drug resistance mutations in katG, rpoB, and embB genes
Source: BMC Infect Dis. 2020 Oct 14;20:752. doi: 10.1186/s12879-020-05479-5 (PMC7557036; doi:10.1186/s12879-020-05479-5)
Supplement: Supplementary file 3 — Additional file 3. [file 12879_2020_5479_MOESM3_ESM.doc]

**Supplementary Table 2**

Drug resistance profile of Beijing genotype of *M. tuberculosis* (n=76)

| **Profile** | **Total** | | **Never treated (n=39)** | | **Previously treated (n=37)** | |
| --- | --- | --- | --- | --- | --- | --- |
|  | **n** | **%** | **n** | **%** | **n** | **%** |
| Pan-drug susceptible | 26 | 34.21 | 18 | 46.15 | 8 | 21.62 |
| Resistant to all tested drugs^*^ | 35 | 44.74 | 11 | 28.21 | 24 | 64.86 |
| Multi-drug resistant | 45 | 59.21 | 17 | 43.59 | 29 | 78.38 |
| Any drug resistance | 50 | 65.79 | 18 | 46.15 | 33 | 89.19 |
| Resistant to INH | 50 | 65.79 | 18 | 46.15 | 33 | 89.19 |
| Resistant to RIF | 47 | 61.84 | 19 | 48.72 | 29 | 78.38 |
| Resistant to EMB | 40 | 52.63 | 11 | 28.21 | 29 | 78.38 |
| Resistant to STR | 37 | 48.68 | 11 | 28.21 | 26 | 70.27 |
| Resistant to INH only | 1 | 1.32 | 1 | 2.56 | 0 | 0 |
| Resistant to RIF only | 1 | 1.32 | 1 | 2.56 | 0 | 0 |
| Resistant to EMB only | 1 | 1.32 | 1 | 2.56 | 0 | 0 |
| Resistant to STR only | 0 | 0 | 0 | 0 | 0 | 0 |
| Resistant to HES | 36 | 47.37 | 11 | 28.21 | 25 | 67.57 |
| Resistant to HER | 36 | 47.37 | 12 | 30.77 | 24 | 64.86 |
| Resistant to ESR | 35 | 46.05 | 12 | 30.77 | 23 | 62.16 |
| Resistant to HSR | 35 | 46.05 | 12 | 30.77 | 23 | 62.16 |
| Resistant to HE | 36 | 47.37 | 11 | 28.21 | 25 | 67.57 |
| Resistant to HS | 36 | 47.37 | 11 | 28.21 | 25 | 67.57 |
| Resistant to ES | 35 | 46.05 | 10 | 25.64 | 25 | 67.57 |
| Resistant to ER | 36 | 47.37 | 11 | 28.21 | 25 | 67.57 |
| Resistant to SR | 35 | 46.05 | 11 | 28.21 | 24 | 64.86 |

*Tested Drugs: Rifampicin, Isoniazid, Ethambutol, Streptomycin
