# Supplementary material for: Detection of Beijing strains of MDR M. tuberculosis and their association with drug resistance mutations in katG, rpoB, and embB genes
Source: BMC Infect Dis. 2020 Oct 14;20:752. doi: 10.1186/s12879-020-05479-5 (PMC7557036; doi:10.1186/s12879-020-05479-5)
Supplement: Supplementary file 2 — Additional file 2. [file 12879_2020_5479_MOESM2_ESM.zip › Supplementary fileR8.doc]

**Selection Criteria:** Most of the samples (~86%) used in this study were part of Dr. Anamika Gupta’s PhD synopsis. Statistical justification of which is described below.

Rest of the samples collected by Dr. Anamika and Dr. Pallavi were also included in the study for better estimate. For statistical calculation of sample size for TB and MDR-TB prevalence previous study of the same department was used. This is the first study focusing on Beijing strain identification from our department with large number of samples.

**Statistical Justification of samples from 2008 to 2012**

**Collection of samples:** 3504 sputum specimens were collected during 2008 – 2012, from the patients of pulmonary tuberculosis, from three TB – centers and analyzed for culture positivity to MTB.

**^#^Statistical Justification:**

To calculate the prevalence rate of tuberculosis (TB) in our region, the finding of the previous study from our department (Unpublished data)* was considered which gives the TB- prevalence of 32%. Based on this value and 5% allowable permissible error the sample size was calculated using the formula stated below. This will provide the estimated 95% confidence limit between 30.4 to 33.6% of TB prevalence.

**Formula:**

**n = 3.96pq/e^2^**

Where, n = number of samples

p = Prevalence of TB

q = 100 – p

e = 5% of the anticipated TB prevalence

n = 3.96 x 32 x 68/ (1.6)^2^

n = 3366 sputum samples

Thus, a sample of 3366 will be required for further investigation of TB prevalence. However, if more samples during process are received that will be taken for the study for better estimate.

To calculate the prevalence rate of multidrug resistant tuberculosis (MDR-TB) in our region, the finding of the previous study from our department (Unpublished data)* was considered which gives the MDR- prevalence of 27% in culture positives. Based on this value and considering the feasibility of experimentation, a bit higher (20%) permissible error was allowed that will provide the estimated 95% limit between 21.6 to 32.4%. So, taking MDR-TB prevalence rate 27% and permissible error 20% of 27% (i.e., 5.4), the sample size was calculated, following above mentioned formula, as below:

n = 3.96 x 27 x 73/ (5.4)^2^

n = 270 culture positive samples

However, the sample size more than 270 was expected among the 3366 sputum samples. So, this, a higher number than 270 culture positives will be considered for better estimate of the prevalence of MDR-TB.

**Details of collection centres:** Three centers from which sputum samples have collected are as follows:

- **Sir Sundar Lal Hospital, Institute of Medical Sciences, Banaras Hindu University, Varanasi:** A tertiary care center, 3384 sputum specimens were collected.
- **Shree Shiv Prasad Gupta District Hospital (Government Hospital), Kabir Chaura, Varanasi:** A secondary care center, 62 sputum specimens were collected.
- **Swami Vivekanand Smarak Rajkiya Chikitsalaya, Bhelupura, Varanasi:** A primary health care unit, 58 sputum specimens were collected.

Samples from both inpatients and out-patients were included.

Of 3504, 328 (9.36%) specimens were culture positive for mycobacteria.
