# Supplementary material for: Detection of Beijing strains of MDR M. tuberculosis and their association with drug resistance mutations in katG, rpoB, and embB genes
Source: BMC Infect Dis. 2020 Oct 14;20:752. doi: 10.1186/s12879-020-05479-5 (PMC7557036; doi:10.1186/s12879-020-05479-5)
Supplement: Supplementary file 2 — Additional file 2. [file 12879_2020_5479_MOESM2_ESM.zip › Supplimentary FileR8.doc]

**Primers to identify Non Beijing Strains of *M. tuberculosis***

F- 5’ AAGCATTCCCTTGACAGTCGAA 3’

R- 5’ GGCGCATGACTCGAAAGAAG 3’

**Non Beijing Strain Sequences**

CTTCGCCATGTCGTCTTGGTCGGTGACAAAGGTCTTGCTCCGATAGCCGGCTCCGCCGCCCAGATAGACA

ATTGGGCCAACTATCGCGTTCACGCCAGGGTACATGGCTCTGTACTCCGCGTAACGCGCCTGATTCACGG

ACGCGGCTGTCTCGGCCAGCGTTTCAAGGAACCGCTCGCCCTCACGCCAGCCGCCGCGAGCGGTGGGACT

GGTGTCGACCACCACGCGGTGCGAGATTGAGGTTCCCGGCGCCAAACATTCCCGGAAGAGCGGCAGGCCA

TCAGGCTTGCCGTGGACATTCATGTCCATCTTCTGGCAGATCAGCAGATCGCTTGTTCTCAGTGCAGGTG

AGTCGGTGACCCTGATCGCCTGAAACAGGTCGTTGACCGCGTCTTGCGGACGGGTGTTGGGGCGCCCCGA

TTTGCGCAACTCCTTCCGCTCAAACCTTTCGCCGTACTGCCGGTGCTCCCGCGTCTGGTGTCCCGGAACA

CGAACAGGTTGGGCCGTCCGCTTATGCACAAGCGACTGCAGGTAGATGCTGCG**AAGCATTCCCTTGACAG**

**TCGA**ACCCGGCACGTAGGGCCTTCCAAGAGGGTCTTTGATGAAAGCGTGAATCTCGTTGAGCGTAAG**CTT**

**CTTTCGAGTCATGCGCC**CGCCTCGACCACGAGATGCACGTCGCGGTTCGATCGACCCGATCTTCACCTCG

TAACCTCGATGCTTAGCAGGATCCAGCTTGACCGCGTTTGGCTCTACCCACTCTTTGAGTGGCGCCGTCG

CCTGTGCCCCATCGGTGTTCATGACGAACGCTTCGAAAGACTTCCTCTTGTGAGCCGGAATGTCTGCGTA

AAGAAGTTCCATGTCCGGGAAGTAGACCCGGTCGCCCTCCACGTGGTACTCCTTCGAGGTCCGCTTCTCG

CCGGATCCGATAAACACCGGCCCCAGGCACCGCAGCGTGAGTTCGAACGGCTTCAGGTAGGTGTTCATGC

GGCGGACTCCGGGAGTGCGAGAAATAGCGGTCGCGCGTAGCTGTAGACCGGATGGTTTCCGCCCAGGCTG

ACGTCGAGGATGCCTCCTTGGAAGGGTCGCGAGAAGACCGAGCCGGCGGCGAATTTGTAGATGTCGCGTT

TGCGCAGGGGCATGTCAGCGTATGTGCTCGACGCGACGAATCCACTGCGCTTGACGAGGCGGTACGTCGC

GCCGGCGAGTGCGGCTTCGAGCTCGTCGTCCGTGGGTAGGGATGTCGTGAGCGTCATCAGACTGGCCGCG

TCGACTGTCGGCGTGAGTGCGGCGGGTGCTTCTGACTCGGTAAGGTTAAACGCTCCGAACCCGCTTGTCC

GTTCGCCGCCCAGCGCGGAGATCCCTTTCAACAGCCTGGTGAGTAGGCCGAGCTCGGACTCGGATCCGGT

CGCCAGCAACCACAGACCCGCGTCCAGCTCGAACCGGAAGTAGCCGACACGGTACGGGTCGGCGTCTTTC

TTTCCGTTGTGGATCGCTGCCTTCGCTGACACGGCGTGGACACCGATCTTGGTCTGCCGCGCCGCGAGTT

CTTTCAGGTCGGCCGTGCCATCGAGGAAGCTGCCAAGCTGGGCAGCGGGAAGAAAGCCGATCTTCTTCGC

CAGCTTCTTCTGCATACTTGAGCCGTCGGACCGAACGCTGTGCAGGGGCTTGGGAACCAGGTAATCGGGC

CCCACATAGGGCAGCAGATCGGTCAACCGCAGCGTCGAGCACGCAACGAGTTCGCCAAGCAGCTGCTGGC

CACCCATCCGTAGCGCTTCAACGCAAAGCGCAGAGTAGAGGGTGTCCGCGGGGCAGCTAATCGTGGACGA

CTCGAGGCCGTGGTCGCCGAAGTGTGTGCGGTCGAAGTCGAACCTAAACAGCCGCGAGTTCATGGTTTAG

CTTCTCCAGCAGAGAACCGTCGAGGGCGCCGACTGCGGCGCGGGCTTTCAGGTTGCTGAACTTGACCTGC

CCGTAGCCACGGGTTCCGCTGCCGCCGAGGTAGTCGAGTTCGAGCAACTTCAGGCCGCGCGCGATGGCGT

TGAAGTCCTCGATGATCTCATCGGAGGAAGGCAGAGACGCCTTCTGTTCCTCGCCGGGGGTGCCGAAGGA

GACCTCGTAGACAAGTGAGAACGCGAACTCGCTGCCGGGGATCACGCGTTCCATCTGGCGAAGGTTTGCC

TTTGCGGTCACCCGGTTGATGGCGTTCTCGAATTTCACCTCGGTGAGAGTCTTAGCGCCGCGGGCTTCGA

GGTCGTCTTTGTTGGTGAGCTTCGTGTCGCGGAAGACGAGTCGGCCCGTCATGTACTCCTCGGTGTCGCC

GAAAAGCCGACGGATATGGGCGTGGTCCTCATTCGGCTTCCTGTAAAACGTTTCTGTGTCGGCGCCGTAT

TGGCGGGACAGCAAGGTGCGGACCTTGCCCTTCAGGCTGGTACCCGGAATCATCGGCAGCCTGCTCAGCG

GATCACGAACGACAGGCTTGTCGACCGCGCCGATGGCGGAGAAGCCATCGCCGGCCCCGATCTGCAGGCC

CGTCAGGACGGTCAGTGTCCCGGTTATCTCGATCTTGGCGTAGCTCGTAGTCATTGGGTTGTCTCACTTG

TCCTTCGGATCGAGGTACTTCTTGTATGCGGCTAGGGCTTCCATGTACCGGCAGAATCGCAGCAGCCCGT

CGCGGCTATCGCCTATCCCTTCCAGCGCTTCTAGGAGTTTCGCGTTTCGGACGAATGTCTTAACCGCGTC

TTCACGCCCGGACTGGTAGACGAACCGGACCCGCAGGTACTGGACCTTCTCCTTCAGCTGACGCGGGAGC

GTGGGGTTGGCGCTCTGCTGCGCCTCGTCGAAGAGCTGTGCGGTCAGGCTGAGTAGCACCCGCAGCTGGG

TTGTGGTCAGCTCGAAGCCGTTCTTTTTCTTTGGCAGGCCGCGAATTACTTCGGCCTGTTTCACATAGTC

GTCTTGGATGACGCTCATTCGGACTCCTCCTTGCGAGTGCGATAGATGTAGAGGTGCAGCGCGGTCTTGA

GTTGCTTGGCGTCTGTCGGATCTTGGAACCATTGGTGTAGCCGGTTAGCAAACTGCTGAAAAGGCGCTGT

GTCACCGGTGGGGTTACGCATGCGCGTGAGGAAGTACACCCATCTGGCCTTTGTGATTCGATCGTCGCGT

TCGGCGAGTAGTTCGAGCAGCTTGTAGATGAAGGCCATGCCGCGTTCTTCGTTGCCACTGAAATAGTCGG

CGATGTGCCGGTACTTCTCCTCGATCACCTTGCTGAGCAGCTCATCCCAGCCGAAGGTGAACTCGCGATC

**AAGCATTCCCTTGACAGTCGA**ACCCGGCACGTAGGGCCTTCCAAGAGGGTCTTTGATGAAAGCGTGAATCTCGTTGAGCGTAAG**CTTCTTTCGAGTCATGCGCC**

**104 bp**
